# Permissive weight bearing versus restrictive weight bearing in surgically treated trauma patients with displaced intra-articular calcaneal fractures (the PIONEER study): study protocol for a multicenter randomized controlled trial

**DOI:** 10.1186/s13063-024-08617-5

**Published:** 2024-11-18

**Authors:** Coen Verstappen, Mitchell L. S. Driessen, Pishtiwan H. S. Kalmet, Lloyd Brandts, Merel Kimman, Michael Edwards, Erik Hermans, Martijn Poeze

**Affiliations:** 1 Department of Trauma Surgery, Maastricht University Medical Center+, P. Debyelaan 25, Maastricht, 6229 HX The Netherlands; 2 Department of Trauma Surgery, Radboud University Medical Center, Geert Grooteplein Zuid 10, Nijmegen, 6525 GA The Netherlands; 3 Department of Clinical Epidemiology and Medical Technology Assessment, Maastricht University Medical Center+, Maastricht, The Netherlands

**Keywords:** Displaced intra-articular calcaneal fractures, Permissive weight bearing, Randomized controlled trial, Trauma patients, Postoperative treatment, Rehabilitation

## Abstract

**Background:**

Following successful treatment, displaced intra-articular calcaneal fractures (DIACFs) necessitate an extensive rehabilitation regimen, significantly influencing functional and socio-economic outcomes. Apart from surgical intervention, the implementation of a comprehensive rehabilitation protocol is crucial to optimize foot stability and functional recovery. The objective of this study is to ascertain the optimal rehabilitation protocol for patients with surgically treated DIACFs, either permissive weight bearing (PWB) or Restricted Weight Bearing, focusing on functional outcomes, health-related quality of life (HRQoL), radiographic parameters, cost-effectiveness, and incidence of complications.

**Methods:**

Study design: A prospective multicenter randomized controlled trial. Study population: Presence of surgically (extended lateral, sinus tarsi, or percutaneous approach) treated unilateral DIACFs (Sanders type II to IV), aged 18–67 years (labor force). Patients must be able to understand and follow weight bearing instructions. N = 115 patients with DIACFs will be included. Interventions: Patients with DIACFs will be randomly allocated to one of the rehabilitation protocols, either PWB or RWB. Primary outcome measure: Functional outcome, measured with the American Orthopaedic Foot & Ankle Society Score (AOFAS)). Secondary outcomes: Functional outcome (Maryland Foot Score, MFS), HRQoL (EuroQol-5D, EQ-5D), differences in radiographic parameters, cost-effectiveness, and complications. Nature and extent of burden: The PWB protocol is aimed to be non-inferior to the RWB protocol. Previous analysis of this protocol in other lower extremity fractures has shown a safe complication rate. Follow-up is standardized according to current trauma guidelines, namely at time points 2, 6, 12 weeks, and 6 months. The radiation exposure for both groups will differ from standard care (one extra CT scan of the foot will be made). Therefore, the burden for participants is considered minimal, with no significant health risks.

**Discussion:**

This study will be the first study to define an optimal rehabilitation regime for surgically treated patients with DIACFs. The limitations of this study include the absence of patient blinding, as this is impossible in rehabilitation. Additionally, the primary outcome measure (AOFAS) has limited validity for DIACFs. However, it is the most commonly used questionnaire in the literature on DIACFs. There is an apparent need since current literature is lacking on this specific topic.

**Trial registration:**

ClinicalTrials.gov NCT05721378, accepted on February 7, 2023.

**Supplementary Information:**

The online version contains supplementary material available at 10.1186/s13063-024-08617-5.

## Administrative information

**Table:** 

**Title {1}**	Permissive Weight Bearing versus Restrictive Weight Bearing in surgically treated trauma patients with displaced intra-articular calcaneal fractures (the PIONEER study): study protocol for a multicenter randomized controlled trial
Trial registration {2a and 2b}	ClinicalTrials.gov ID: NCT05721378
Protocol version {3}	Version 6.5 (April 1, 2024)
Funding {4}	- Orthopaedic Trauma Association (OTA) grant number 7956 - Osteosynthesis and Trauma Care Foundation (OTCF) grant number: 2023-MPCV
Author details {5a}	1. Department of Trauma Surgery, Maastricht University Medical Center + , P. Debyelaan 25, 6229 HX, Maastricht, The Netherlands 2. Department of Trauma Surgery, Radboud University Medical Center, Geert Grooteplein Zuid 10, 6525 GA, Nijmegen, The Netherlands 3. Department of Clinical Epidemiology and Medical Technology Assessment, Maastricht University Medical Center + , Maastricht, The Netherlands
Name and contact information for the trial sponsor {5b}	Maastricht University Medical Center, Clinical Trial Center Maastricht (CTCM). Oxfordlaan 70, 6229 EV, Maastricht, the Netherlands www.ctcm.nl
Role of sponsor {5c}	The sponsors and funder do not have any influence on study design, data collection, management, analysis, or interpretation of data. The funding sources have no influence on the decision to submit for publication

### Introduction

#### Background and rationale {6a}

Fractures of the calcaneus constitute up to 2% of all fractures and account for 60% of all fractures of the foot. Among calcaneal fractures, approximately 65% are intra-articular [1]. Surgical intervention is frequently required to restore the subtalar joint and the anatomical shape of the calcaneus. Given that calcaneal fractures predominantly affect middle-aged males, their socio-economic ramifications are considerable [2–5]. Approximately 20% of patients will not return to work within 1 year after injury [6]. Hence, post-fracture rehabilitation plays a pivotal role in the management of calcaneal fractures.

“The PermIssive Or Non wEight bEaRing in calcaneal fractures study” (the PIONEER study) will be the first prospective randomized controlled trial (RCT) comparing two rehabilitation protocols. Increasing evidence suggests that early weight bearing protocols such as permissive weight bearing (PWB), reduce the time to full weight bearing without adversely affecting patient-reported quality of life, pain levels, or complication rates [7, 8]. The greatest benefits of early weight bearing in lower extremity fractures are believed to be realized within the initial 6 months post-surgery [9]. Additionally, a recent systematic review on calcaneal fractures indicates that Early Weight Bearing (EWB) may not lead to inferior outcomes compared to the current, more conservative, weight bearing regimes [10]. Presently, both non-weight bearing (NWB) and EWB protocols are utilized in Dutch hospitals for calcaneal fractures [11]. However, a prospective study comparing these two rehabilitation protocols in displaced intra-articular calcaneal fractures (DIACFs) is lacking. Furthermore, there is a paucity of research comparing complication rates between the two protocols in the aftertreatment phase of DIACFs. Consequently, the safety of EWB following surgery in patients with DIACFs remains uncertain.

**Fig. 1:**
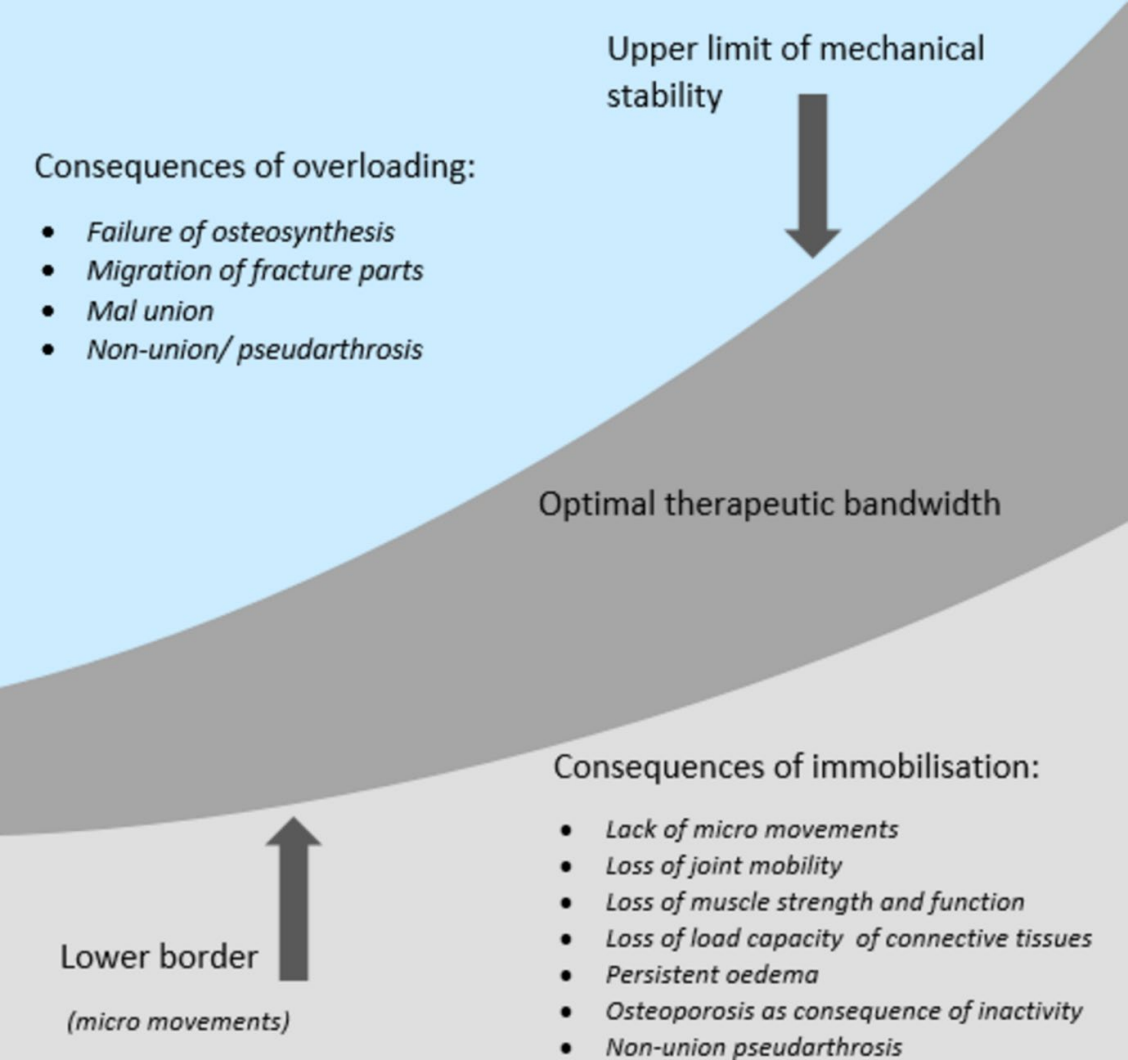
Schematic overview of the consequences of loading on the consolidation

In postoperative fracture management, it is well-established that both over- and underloading can hinder recovery. As depicted in Fig. 1, it is hypothesized that maintaining a balance between a certain level of weight bearing and periods of partial inactivity is crucial for successful rehabilitation of trauma patients [12]. Therefore, the goal of aftertreatment in operatively treated trauma patients should be to prevent issues stemming from exceeding the upper and/or lower thresholds of weight bearing (i.e., overloading and immobilization). Literature confirms that inadequate loading may result in complications such as persistent edema, osteoporosis due to inactivity, reduction in joint mobility, muscle strength, functional capacity, and connective tissue load-bearing capacity [13]. Conversely, excessive loading can lead to failure of osteosynthesis and migration of fracture parts, subsequently resulting in malunion or nonunion [14]. Simultaneously, a certain minimum level of loading is necessary to induce micromovements between fractured bone components and initiate biological processes that facilitate fracture healing and counteract the effects of immobilization, such as muscle mass loss [15].

Currently, the AO (*Arbeitsgemeinschaft für Osteosynthesefragen*) protocol for postoperative management of DIACFs involves NWB for 8 to 12 weeks, followed by a weekly 25% increment in weight loading [16]. Despite the well-established positive impact of early weight bearing on fracture healing and preservation of muscle and bone mass, this regimen has remained the standard for decades [17]. Consequently, there arises a question as to whether physicians and physiotherapists may be overly cautious, driven by concerns about secondary fracture displacement or mechanical construct failure.

### Objectives {7}

The purpose of ‘the PIONEER study’ is to conduct a randomized controlled trial in surgically treated patients with DIACFs who undergo rehabilitation with either PWB or Restricted Weight Bearing (RWB). The primary aim of the study is to explore functional outcomes for surgically treated patients with DIACFs and will be allocated to one of the two distinct rehabilitation protocols (PWB or RWB) over a 6-month period. Secondary objectives include comparing disparities in additional functional outcomes and health-related quality of life (HRQoL). Additionally, variances in radiographical parameters (Böhlers angle and posterior facet joint alignment), cost-effectiveness, and complication rates will be assessed and compared.

### Trial design {8}

The PIONEER study will be a prospective multicenter RCT conducted in the Netherlands, with parallel groups, allocation ratio 1:1. The primary outcome measure will be evaluated based on the principles of non-inferiority. Secondary outcomes on superiority.

### Economic aspects

Globally, healthcare systems are grappling with escalating costs, heightening the imperative for efficiency in healthcare delivery. Consequently, it becomes crucial to optimize the utilization of public resources [18, 19]. Hence, a cost-effectiveness analysis will be conducted in the present study. To the best of our knowledge, no prior studies have explored the cost-effectiveness of Permissive Weight Bearing (PWB) versus Restricted Weight Bearing (RWB) in surgically treated patients with DIACFs.

### Evaluation of treatment

To date, one systematic review has compared the impact of time to post-operative weight bearing on functional and clinical outcomes in adults with DIACFs across 72 studies [10]. The sole Patient-Related Outcome Measure (PROM) examined in this review is the American Orthopaedic Foot and Ankle Society (AOFAS) score. According to these findings, EWB does not appear to lead to impaired functional outcomes when compared to more conservative weight bearing regimes [10]. However, the studies included in the comparison were categorized based on weight bearing, and no direct comparison of predetermined weight bearing regimes was conducted.

In recent years, the importance of healthcare efficiency has grown significantly. It is imperative to examine patient healthcare costs as well as costs to families and society for both treatment options. Surprisingly, these costs have not been explored in previous studies. This study will be the first prospective RCT to investigate these costs associated with both rehabilitation regimens.

## Methods: participants, interventions, and outcomes

### Study setting {9}

The PIONEER study will be structured as a prospective, randomized controlled, multicenter trial. It will be conducted in 11 medical institutions across the Netherlands, both urban and academic hospitals. All hospitals joined another study together and were asked to participate in the PIONEER study. Details regarding the locations of participating medical institutions are shown in Additional file 1. Both rehabilitation protocols will be available in all hospitals.

The study will adhere to the SPIRIT (Standard Protocol Items: Recommended for Interventional Trials) 2013 statement. Approval from the local medical ethics committee has been secured, with registration number: NL83269.068.23. Additionally, ‘the PIONEER study’ is registered in the ClinicalTrials.gov database under registration number: NCT05721378 as of 07/02/2023 and will be conducted in accordance with the principles outlined in the Declaration of Helsinki.

Over the course of a 1-year period, patients will be enrolled. Enrolment is commenced on July 1st, 2024. The participating institutions in the PIONEER study are specified in the Additional file 1.

### Eligibility criteria {10}

The study population comprises patients with surgically treated DIACFs, for whom weight bearing is prohibited for 8 to 10 weeks post-operatively in accordance with current guidelines. Specifically targeting individuals in the labor force, the inclusion criteria encompass patients aged between 18 and 67 years old, facilitating the estimation of reductions in healthcare and societal costs. Eligible patients must possess the capacity to comprehend and adhere to instructions. Written informed consent will be obtained from all participants before their inclusion in the study.

#### Inclusion criteria

- Surgically treated (e.g., extended lateral, sinus tarsi, and percutaneous approach) trauma patients with isolated unilateral DIACFs, less than 6 weeks after the initial trauma, Sanders type II–IV [20]
- Isolated unilateral calcaneal fractures
- Age between 18 and 67 years old (labor force)
- Being able to understand the questionnaires and measurement instructions
- Indication for open/closed reduction and internal fixation
- Written Informed Consent

#### Exclusion criteria

- Acute or existing amputation (upper limb, lower limb, feet)
- Open calcaneal fractures (excluding medial wound without compromising surgical approach)
- Unable to comply to the PWB protocol due to pre-existing conditions of the arms and legs (e.g. unable to use crutches due to hemiparalysis)
- Severe non-fracture-related comorbidity of the lower extremity
- Pre-existent immobility (loss of muscle function of one or both legs)
- Dependent in activities of daily living (e.g. due to dementia, Alzheimer, New York Heart Association class IV angina, heart failure, or oxygen-dependent chronic obstructive pulmonary disease)
- Rheumatoid arthritis of the lower extremities
- Severe psychiatric comorbidities that lead to the inability to comply with the treatment protocol
- Pathologic fractures (metastasis, secondary osteoporosis)
- Peripheral neuropathy and/or diabetes
- Alcohol- or drug abuse preventing adequate follow-up
- Primary indication for arthrodesis subtalar joint

### Who will take informed consent? {26a}

The physician, who holds clinical responsibility for the patient, will bring the study to the patient's attention. If necessary, the physician will provide the patient with an information folder about the study and contact the investigator to discuss inclusion criteria. Due to the inherent dependency relationship between the doctor and patient, a Local Investigator (LI) will be assigned in each hospital. The LI will explain the study to the patients, providing them with a detailed patient information letter and verbal explanation, and then seek their willingness declaration. This interaction will only occur after the patient has given permission to be approached by the investigator.

Patients will be given 1 week postoperatively to decide whether to participate. The consideration period will commence once the patients receive the patient information folder and the oral explanation. Signing the informed consent letter will only occur after patients have had the opportunity to ask any additional questions, ensuring their understanding of participation. If patients agree to participate, the informed consent letter will be completed in the presence of the investigator and stored at the investigation site.

### Additional consent provisions for collection and use of participant data and biological specimens {26b}

Not applicable; no biological specimens will be collected.

## Interventions

### Explanation for the choice of comparators {6b}

The absence of prospective studies on weight bearing protocols prevents the favoring of one rehabilitation regimen (PWB or RWB) over the other in the treatment of surgically managed trauma patients with DIACFs. Consequently, there is a clear need for a large-scale prospective, high-quality RCT to evaluate whether the PWB protocol yields improved functional outcomes, radiographical parameters, enhanced quality of life, and reduced costs. Such a trial should also examine cost-effectiveness and complications, as cost considerations could be pivotal in decision-making, particularly when both rehabilitation regimens yield comparable PROMs.

### Intervention description {11a}

#### Permissive weight bearing

The PWB protocol represents a relatively new approach to early weight bearing, enabling earlier postoperative weight bearing. Progression of weight bearing is determined by the patient’s subjective experience, such as pain levels and weight bearing tolerance, along with the clinical expertise of the treating physician and physiotherapist, in line with the PROMETHEUS protocol [12]. The rehabilitation regimen commences with 2 weeks of immobilization to facilitate wound healing. Subsequently, patients are allowed to bear as much weight as tolerated, guided by their pain levels and comfort [12].

#### Restrictive weight bearing

The RWB protocol adheres of 8 to 12 weeks of postoperative weight bearing restriction, in the range of 0–10%, so-called *touching load*, as outlined in the current AO Guidelines [16]. Following this initial phase, which entails restricted weight bearing, a gradual increase in weight bearing is implemented. This entails a weekly increment of 25% over the course of 4 weeks. During each patient-physician encounter, the weight bearing advice provided to the patient is meticulously recorded. This includes whether the recommendation is for unloaded, partial weight bearing with clarification, or full weight bearing. Furthermore, any additional advice imparted to the patient is also documented.

### Criteria for discontinuing or modifying allocated interventions {11b}

In accordance with Sect. 10, subsection 4 of the WMO, the sponsor will suspend the study if there is sufficient reason to assume that continuing the study would compromise the health or safety of the subjects. The sponsor will promptly notify the accredited Medical Ethical Board of any temporary suspension, providing the reasons for such action without undue delay. The study will remain suspended until a further (positive) review from the accredited Medical Ethical Board is obtained. Throughout this process, the Coordinating Investigator (CI) will ensure continuous communication with the enrolled subjects.

Besides, the LI or CI reserves the right to withdraw a participant from the study due to urgent medical reasons. Participants retain the autonomy to withdraw from the study at any point for any reason, without facing any repercussions. Data collected up until the time of withdrawal will be utilized for study purposes.

### Strategies to improve adherence to interventions {11c}

This is not applicable to our study, as strategies to improve adherence to intervention protocols and procedures for monitoring adherence are not required.

### Relevant concomitant care permitted or prohibited during the trial {11d}

There are no relevant restrictions on concomitant care or interventions during this study. All standard concomitant care is permitted.

### Provisions for post-trial care {30}

The sponsor maintains liability insurance in accordance with article 7 of the Medical Research Involving Human Subjects Act (WMO). Aftertreatment following the PWB protocol is already implemented in several hospitals in the Netherlands, including for DIACFs. While no adverse effects have been noted from clinical experience, there may be potential risks that are currently unknown. Additionally, a recent retrospective study, not yet published, and a systematic review and meta-analysis reported no negative effects on patients’ reported quality of life, pain, or complication rates.

### Outcomes {12}

#### Primary outcome

The primary outcome is the functional outcome score, defined by the American Orthopaedic Foot & Ankle Society (AOFAS). The AOFAS score is a clinician-based assessment tool that integrates both subjective and objective information. Patients provide self-reports on their pain levels, while physicians evaluate alignment. This scoring system is intended to assist physicians in standardizing patient assessments, thereby facilitating the comparison of study results with previous data. Scores on the AOFAS scale range from 0 to 100, with a score of 100 points indicating optimal health for the foot and ankle [21]. Time points: measured at 2 weeks, 6 weeks, 12 weeks, and 6 months post-surgery.

#### Secondary outcomes

Secondary outcomes encompass several measures.

1. The Maryland Foot Score (MFS): The MFS is a tool for assessing foot disorders, comprising evaluations of pain, gait, functional activities, and cosmesis. Scores range from 0 to 100 points, with < 50 indicating poor, 50–74 as fair, 75–89 as good, and 90–100 as excellent outcomes [22]. Time points: collected at 2 weeks, 6 weeks, 12 weeks, and 6 months post-surgery.
2. Health-related quality of life (HRQoL) using the EuroQoL 5-Dimension 5-Level (EQ-5D-5L): This self-administered questionnaire comprises five dimensions (mobility, self-care, daily activities, pain/discomfort, and depression/anxiety), each rated on five levels. Utilities calculated using preferences from the Dutch tariff will enable Quality Adjusted Life Years (QALY) scoring, besides the questionnaire is recommended by the Dutch guidelines [23, 24]. Time points: collected at baseline, 2 weeks, 6 weeks, 12 weeks, and 6 months post-surgery.
3. Radiographical characteristics: Evaluation of Böhlers angle and posterior facet joint alignment via radiographic assessment by a blinded radiologist will be conducted at scheduled physician visits. CT scans early post-operatively and at 6 months will be used to measure differences in Böhlers angle and posterior joint alignment [25]. Differences in the Böhlers angle and posterior joint alignment (Δ = CT_6months_—CT_post-op._) will be calculated. Normal Böhlers angle ranges between 25° and 40° [26]. Intra-articular step-off or gap < 2 mm of the posterior facet is considered well-reduced [3]. Time points: early post-operative (‘baseline’) and at 6 months post-surgery CT scan.
4. Medical consumption and productivity: Measured using the Medical Consumption Questionnaire (iMCQ) and Productivity Costs Questionnaire (iPCQ). The iMCQ assess healthcare utilization, while the iPCQ captures productivity losses. The iPCQ and iMCQ are generic and complimentary questionnaires, that can be used in every indication and are adapted to the context of this study [27]. Time points: collected at baseline, 12 weeks, and 6 months post-surgery.
5. Total complication rate: A wound infection is defined as (but not limited to) purulent wound drainage, inflammation, erythema, fever, increased white blood cell count, increased C-reactive protein and/or increased erythrocyte sedimentation rate, necessitating admission for antibiotic treatment and/or revision surgery for infection.

A dislocation is defined as the migration of fracture parts, defined as >3 mm articular step-off or gap and/or varus/valgus malalignment > 5° after weight bearing. A non-union is defined as no radiographic union achieved after 6 months or no progress in healing. Failure of osteosynthesis is defined as loosening or breakage of implants, including the rate of reoperation (e.g., leading to removal of material, arthrodesis). Postoperative arthritis will be screened on CT scans. Time points: evaluated at 2 weeks, 6 weeks, 12 weeks, and 6 months post-surgery.

### Participant timeline {13}

**Fig. 2:**
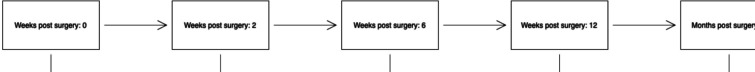
Overview of the measurements

Regular follow-up will be performed for both weight bearing regimes. Appointments will be scheduled at 0, 2, 6, and 12 weeks, and at 6 months (Fig. 2).

### Sample size {14}

To our knowledge, there is no literature available that directly compared our primary outcome (AOFAS Score) in PWB versus RWB for surgically treated patients with DIACFs. Therefore, unfortunately, the MCID is unknown for the AOFAS Score [28, 29].

However, the estimated minimal clinical difference (MCID) can be calculated as one half of the standard deviation (0.5 SD) [30]. Preliminary results of a systematic review that is currently performed by our group, found a mean AOFAS Score of 83.7 (SD 8.5) for the RWB group. 0.5 × 8.5 results in a MCID of 4.3, respectively.

Besides, a recent Cochrane review reported a MCID for the AOFAS Score in other foot conditions that range from 2.0 to 7.9 [29]. This results in a mean MCID of (2.0 + 7.9)/2 = 5, respectively.

For this sample size calculation, a MCID of 5 points was assumed, approximating a 0.5 SD of the estimated mean AOFAS score after RWB for surgically treated patients with DIACFs [30].

In order to be able to detect such a difference between the two groups, assuming that an equal number of patients in both groups will be included, with an alpha of 5% and beta of 20% (power 80%), a pooled SD of 8.5 and a true difference of zero points between the RWB and PWB group the sample size should be at least 46 for both groups = 92 in total. Anticipating a 20% drop out a total of 115 patients must be recruited for this study. The sample size calculation was performed with a two-sample mean test for non-inferiority using the TrialSize package within “R.”

### Recruitment {15}

Each medical institution participating in “the PIONEER study” will appoint a Local Investigator (LI). The Coordinating Investigator (CI) will oversee the trial and provide guidance to all LIs across each medical institution. Together, the LIs (comprising trauma or orthopedic surgeons) and the CI will constitute “the PIONEER study” group. The LIs will actively screen patients for eligibility at their institution’s emergency department or outpatient clinic. In cases where there is uncertainty regarding the eligibility criteria, the LI will communicate with the CI, who will make the final decision.

## Assignment of interventions: allocation

### Sequence generation {16a}, concealment mechanism {16b} and implementation {16c}

After recording patient characteristics from the electronic medical records, patients will be randomly assigned to one of the two aftertreatment protocols in a 1:1 ratio. Randomization will be conducted using a web-based computer system (Castor EDC) developed by MEMIC (the center for data and information management at the Faculty of Health, Medicine, and Life Sciences of Maastricht University and MUMC +). Block randomization with random permuted block sizes will be employed. If a replacement patient is needed, they will be re-randomized following the same conditions.

## Assignment of interventions: blinding

### Who will be blinded {17a}

Blinding for the type of aftertreatment will not be possible. However, in Castor EDC, randomization rights will be set up correctly to ensure that all users who need to be blinded do not have access to the allocation data. Only users with the “view randomization” right, will be able to see to which group a participant (record) has been allocated; so-called *concealed allocation*.

### Procedure for unblinding if needed {17b}

This is not applicable as blinding is not feasible for this study. Consequently, there is no need for an unblinding procedure.

## Data collection and management

### Plans for assessment and collection of outcomes {18a}

Baseline characteristics will be retrieved by the LI from the electronic patient file. A clinical assessment will be conducted by the LI and recorded in the case report form using the online data management system Castor. Subsequent follow-ups will involve screening the electronic patient file to evaluate any late complications, re-interventions, re-admissions, duration of medical institution stay, and consultations at the medical institution. The LI of the medical institution or, if necessary, the CI will carry out this screening and follow-up assessment. All data collected will also be documented in the case report form by either the LI or CI. In the event of any queries regarding the data, the CI will be contacted by the LI or site investigator.

Additionally, the patients will be administered questionnaires at 0, 2, 6, and 12 weeks, as well as 6 months post-surgery, irrespective of the rehabilitation protocol.

### Plans to promote participant retention and complete follow-up {18b}

To promote participant retention and ensure complete follow-up, LIs and CIs will call patients who do not complete their questionnaires. This approach aims to maximize data collection and maintain the integrity of the study outcomes.

### Data management {19} and confidentiality {27}

All questionnaires will be distributed and completed digitally. Enrolled patients will receive a link via email or SMS, facilitated by Castor, an electronic data capture and management application [31]. Castor is a validated system and approved by external auditors and complies with relevant laws and regulations, including ICH E6 Good Clinical Practice, 21 CFR Part 11, European Union Annex 11, General Data Protection Regulation, HIPAA (US), ISO 9001 and ISO 27001 [31].

In addition to standard care, preoperative and postoperative (prior to initial mobilization) a CT scan of the injured calcaneus will be conducted. An additional CT scan will be performed at 6 months postoperative. Subsequent radiographic follow-up will involve X-rays as indicated, also in accordance with standard care. Interpretation of CT scans and X-rays will encompass evaluation of Böhlers angle alignment, posterior facet joint alignment (including step-off and gap assessment), and identification of posttraumatic arthritis.

### Plans for collection, laboratory evaluation, and storage of biological specimens for genetic or molecular analysis in this trial/future use {33}

This is not applicable, as there are no plans for the collection, laboratory evaluation, or storage of biological specimens for genetic or molecular analysis in this trial or for future use in ancillary studies.

## Statistical methods

### Statistical methods for primary and secondary outcomes {20a}

Baseline patient characteristics will be presented according to the by aftertreatment regime. Continuous variables will be summarized using either mean and standard deviation or as median and interquartile range. Categorical variables will be summarized as count and percentages.

All analyses of the primary and secondary study parameters will be conducted following both the intention-to-treat principle and the per-protocol principle. Statistical significance will be determined using a *p*-value of ≤ 0.05. All analyses will be carried out using “R” or IBM SPSS version 28 or later.

#### Primary study parameter

For the primary research question, the AOFAS Score at 6 months, a continuous outcome, will be utilized. The relative effectiveness of PWB compared to RWB on the AOFAS score will be analyzed using a linear regression model. The model will be adjusted for the type of surgery and hospital. The objective is to assess the non-inferiority of PWB compared to RWB, with a non-inferiority margin set at 5 points. To evaluate non-inferiority, a two-sided hypothesis test will be conducted to determine whether the lower limit of the confidence interval for the estimated treatment effect exceeds the predefined non-inferiority margin. The corresponding p-value for non-inferiority will be calculated using the input from the estimated model. The assumptions for the linear regression model will be evaluated, including linearity, independence of errors, homoscedasticity, and normality of residuals.

#### Secondary study parameters

The MFS score at 6 months will be analyzed using a linear regression model to compare between the PWB and RWB groups, similar to the analysis of the primary study parameter. EQ-5D scores at 6 months will be compared between groups using a linear mixed-effects regression model to account for clustering of multiple observations within each patient. Both absolute differences between groups, as well as the difference in slope over time will be estimated. All parameters will be reported, including 95% confidence interval. Binary secondary outcome variables (i.e., having received any secondary surgical procedure, experienced any type of complication, showing differences on radiographs) will be compared between groups using logistic regression analysis.

### Interim analyses {21b}

Not applicable; there will be no interim analysis conducted.

### Methods for additional analyses {20b}

Economic evaluation: a cost-effectiveness analysis (CEA) will be performed to assess whether PWB is value for money. Alongside the trial we will collect individual-level data on intervention costs, healthcare costs, patient and family costs, and productivity costs, using an adapted version of the iMCQ and the iPCQ. Costs will be calculated by multiplying volumes (resource use) with unit costs. For the unit costs, we will use the Dutch costing guidelines [32, 33]. Cost prices will be expressed in euros. If necessary, existing cost prices will be updated to 2024 using the consumer price index [23]. Following the Dutch guidelines, an annual discount rate of 1.5% will be applied for the effects, and future costs will be discounted to their present value by a rate of 4% [23]. The Incremental cost-effectiveness ratio (ICER) will be determined based on incremental costs and effects of PWB versus RWB. The robustness of the ICER will be checked by non-parametric bootstrapping. The bootstrapped cost-effectiveness ratios will be subsequently plotted in a cost-effectiveness plane, in which the vertical line reflects the difference in costs and the horizontal line reflects the difference in effectiveness. The bootstrapped ICERs will also be depicted in a cost-effectiveness acceptability curve showing the probability that permissive weight bearing protocol and AO guidelines is cost-effective using a range of ceiling ratios. Additionally, to demonstrate the robustness of our base-case findings a multi-way sensitivity analysis will be performed. In the sensitivity analysis, uncertain factors of assumptions in the base case analysis will recalculated in order to assess whether the assumptions have influenced the ICER, for example by varying cost prices and volumes between minimum and maximum [34].

### Methods in analysis to handle protocol non-adherence and any statistical methods to handle missing data {20c}

Missing data will be addressed through stochastic regression imputation. In addressing protocol non-adherence, the analysis will be conducted on an intention-to-treat (ITT) basis, including all participants as originally assigned regardless of their adherence to the study protocol. For handling missing data, multiple imputation techniques will be employed to ensure the robustness and validity of the statistical analyses.

### Plans to give access to the full protocol, participant-level data and statistical code {31c}

Plans to grant public access to the full protocol, participant-level dataset, and statistical code are as follows: The full protocol will be made available upon request. Access to the participant-level dataset and statistical code will be granted to qualified researchers upon reasonable request and after appropriate data use agreements are in place to ensure the confidentiality and integrity of the data.

## Oversight and monitoring

### Composition of the coordinating center and trial steering committee {5d}

During this study, oversight and direction will be provided by the Principal Investigator. The Coordinating Investigator There is no Trial Steering Committee or Stakeholder and Public Involvement Group. The Coordinating Investigator will serve as the primary liaison between the various local investigators and the Principal Investigator. The Principal Investigator and Coordinating Investigator will meet every 2 to 3 weeks to discuss study progress, address challenges, and make any necessary adjustments to the protocol. The entire study team, including local investigators, will convene approximately once per quarter to review overall study progress, address any ongoing issues, and ensure alignment with study objectives and regulatory requirements. Additionally, these meetings will provide an opportunity to review safety data, monitor adherence to protocol, and ensure quality control across study sites.

### Composition of the data monitoring committee, its role and reporting structure {21a}

In consultation with the Medical Ethics Review Committee, it has been determined that establishing a Data Monitoring Committee is not necessary.

### Adverse event reporting and harms {22}

An adverse event (AE) encompasses any undesirable or unwanted symptom or sign to experienced by an enrolled subject during the study, irrespective of whether it is associated with an investigational intervention during the clinical trial. AEs may be reported by the subject, observed by the investigator, or recorded by the staff. Both serious adverse events (SAEs) and AEs will be documented in a trial file managed by the Clinical Trial Center Maastricht (CTCM). The investigator will report all SAEs to the sponsor without undue delay after obtaining knowledge of the events. The sponsor will report the SAEs through a web portal to the accredited ethical committee, within 7 days of first knowledge for SAEs that result in death or are life-threatening followed by a period of a maximum of 8 days to complete the initial preliminary report. All other SAEs will be reported within a period of a maximum of 15 days after the sponsor has first knowledge of the serious adverse events.

### Frequency and plans for auditing trial conduct {23}

On beforehand and during the trial, after every five patients per center, the process will be independently audited by the CTCM.

### Plans for communicating important protocol amendments to relevant parties (e.g., trial participants, ethical committees) {25}

If an amendment to the protocol is required, the protocol will be updated accordingly, and the amendment will be reported to the sponsor. Subsequently, the amendment must be reviewed and approved by the METC before implementation. After METC approval, all participating centers will be informed of the changes to ensure consistent application across study sites. Any deviations from the protocol will be fully documented using a breach report form to maintain comprehensive records of all protocol deviations. Additionally, any amendments will be reflected in the ClinicalTrials.gov registry, with the record updated to capture the latest protocol modifications. The trial is registered in ClinicalTrials.gov, ID: NCT05721378.

### Dissemination plans {31a}

Publication will adhere to internationally recognized scientific and ethical standards concerning publications and authorship, including the Uniform Requirements for Manuscripts Submitted to Biomedical Journals set forth by the International Committee of Medical Journal Editors (ICMJE). In addition, the ClinicalTrials.gov registry will be updated with the study results to ensure transparency and accessibility. Study data will not be made available in an open data repository; however, key findings will be shared and presented at relevant scientific conferences to contribute to the broader medical and scientific community. Upon completion of the study, patients will be informed of the results through a lay summary, ensuring they receive accessible information regarding the outcomes and relevance of the study.

## Discussion

The current paper outlines the protocol for a multicenter randomized controlled trial to be conducted in 11 hospitals across the Netherlands. It provides comprehensive details on the study’s objectives, methodology, including patient flow, randomization procedures, recruitment strategies, rehabilitation protocols, and data analysis methods. Additionally, it offers guidance for presenting and publishing the results. Notably, this study will be the first to establish an optimal rehabilitation regimen for patients with DIACFs, and it possesses several strengths. Firstly, it adopts a randomized controlled trial design, widely regarded as the gold standard in clinical research. The involvement of multiple Dutch hospitals enhances the generalizability of the findings. Moreover, this design minimizes potential biases inherent in observational studies, facilitating a more accurate assessment of intervention effectiveness.

However, several limitations should be acknowledged. Firstly, the inevitable heterogeneity in surgical treatment across participating centers may impact protocol consistency and consequently, outcomes. Standardized protocols and staff training will address this concern. Secondly, compliance bias may arise as patients may not strictly adhere to allocated weight bearing regimes. Again, standardized protocols and staff training will mitigate this bias. Thirdly, patient blinding is unfeasible for rehabilitation, potentially compromising methodological rigor and introducing bias into results. However, blinding will be implemented for radiological evaluations. Additionally, the 1-year follow-up period limits assessment of longer-term outcomes, although the greatest effect of early weight bearing is typically observed within the first 3 to 6 months post-surgery.

In conclusion, existing retrospective literature provides inadequate methodological data to determine the superiority of one rehabilitation protocol over another. The PIONEER study will offer evidence on whether to favor one weight bearing protocol over another and will ascertain the safety and efficacy of these protocols for patients with DIACFs.

## Trial status

Current protocol number: 6.5 (April 1, 2024). Expected study start: July 1, 2024. Expected date of complement of the recruitment: July 1, 2025.

## Supplementary Information

Additional file 1. Participating institutions in ‘the PIONEER study’ in alphabetical order.

## Data Availability

All data generated or analyzed during this study are included in this published article.

## Abbreviations

AO: Working group for bone fusion issues; in German: Arbeitsgemeinschaft für Osteosynthesefragen
AOFAS: American Orthopaedic Foot & Ankle Society
Castor EDC: Castor electronic data capture
CEA: Cost-effectiveness analysis
CFR: Code of Federal Regulations
CI: Coordinating Investigator
CTCM: Clinical Trial Center Maastricht
CT scan: Computed tomography scan
DIACF: Displaced Intra-Articular Calcaneal Fracture
EQ-5D: EuroQoL 5-Dimension 5-Level
EWB: Early weight bearing
HIPAA: Health Insurance Portability and Accountability Act
IBM: International Business Machines Corporation
ICER: Incremental cost-effectiveness ratio
ICH: International Council for Harmonisation of Technical Requirements for Registration of Pharmaceuticals for Human Use
ISO: International Organization for Standardization
iMCQ: Medical Consumption Questionnaire
iPCQ: Productivity Cost Questionnaire
LI: Local Investigator
METC/MREC: Medical research ethics committee (MREC); in Dutch: Medisch Ethische Toetsing Commissie (METC)
MFS: Maryland Foot Score
MUMC +: Maastricht University Medical Center +
NWB: Non-weight bearing
OTA: Orthopaedic Trauma Association
OTCF: Osteosynthesis and Trauma Care Foundation
PROM: Patient-reported outcome measure
PWB: Permissive weight bearing
QALY: Quality adjusted life year
(HR)QoL: (Health-related) quality of life
RCT: Randomized controlled trial
RWB: Restricted weight bearing
(S)AE: (Serious) adverse event
SMS: Short Message Service
SD: Standard deviation
SPIRIT: Standard Protocol Items: Recommended for Interventional Trials
SPSS: Statistical Package for the Social Sciences
US: United States of America
WMO: Medical Research Involving Human Subjects Act (in Dutch: Wet Medisch-wetenschappelijk Onderzoek met Mensen)
