# Supplementary material for: Permissive weight bearing versus restrictive weight bearing in surgically treated trauma patients with displaced intra-articular calcaneal fractures (the PIONEER study): study protocol for a multicenter randomized controlled trial
Source: Trials. 2024 Nov 18;25:778. doi: 10.1186/s13063-024-08617-5 (PMC11572059; doi:10.1186/s13063-024-08617-5)
Supplement: Supplementary file 1 — Additional file 1. Participating institutions in ‘the PIONEER study’ in alphabetical order. [file 13063_2024_8617_MOESM1_ESM.docx]

Additional file 1 – participating institutions in ‘the PIONEER study’ in alphabetical order

Alrijne Hospital, Leiderdorp

Amphia Hospital, Breda

Amsterdam University Medical Center

Catharina Hospital, Eindhoven

Elisabeth-TweeSteden Hospital, Tilburg

Haaglanden Medisch Centrum, Den Haag

Maastricht University Medical Center

Maasstad Hospital, Rotterdam

Radboud University Medical Center, Nijmegen

Rijnstate Hospital, Arnhem

Zuyderland Medical Center Parkstad, Sittard-Geleen/Heerlen
